# Diversity of a cytokinin dehydrogenase gene in wild and cultivated barley

**DOI:** 10.1371/journal.pone.0225899

**Published:** 2019-12-05

**Authors:** Beata I. Czajkowska, Conor M. Finlay, Glynis Jones, Terence A. Brown

**Affiliations:** 1 Department of Earth and Environmental Sciences, Manchester Institute of Biotechnology, University of Manchester, Manchester, England, United Kingdom; 2 Lydia Becker Institute of Immunology and Inflammation, School of Biological Sciences, University of Manchester, Manchester, England, United Kingdom; 3 Department of Archaeology, University of Sheffield, Northgate House, West Street, Sheffield, England, United Kingdom; Murdoch University, AUSTRALIA

## Abstract

The cytokinin dehydrogenase gene *HvCKX2*.*1* is the regulatory target for the most abundant heterochromatic small RNAs in drought-stressed barley caryopses. We investigated the diversity of *HvCKX2*.*1* in 228 barley landraces and 216 wild accessions and identified 14 haplotypes, five of these with ten or more members, coding for four different protein variants. The third largest haplotype was abundant in wild accessions (51 members), but absent from the landrace collection. Protein structure predictions indicated that the amino acid substitution specific to haplotype 3 could result in a change in the functional properties of the HvCKX2.1 protein. Haplotypes 1–3 have overlapping geographical distributions in the wild population, but the average rainfall amounts at the collection sites for haplotype 3 plants are significantly higher during November to February compared to the equivalent data for plants of haplotypes 1 and 2. We argue that the likelihood that haplotype 3 plants were excluded from landraces by sampling bias that occurred when the first wild barley plants were taken into cultivation is low, and that it is reasonable to suggest that plants with haplotype 3 are absent from the crop because these plants were less suited to the artificial conditions associated with cultivation. Although the cytokinin signalling pathway influences many aspects of plant development, the identified role of *HvCKX2*.*1* in the drought response raises the possibility that the particular aspect of cultivation that mitigated against haplotype 3 relates in some way to water utilization. Our results therefore highlight the possibility that water utilization properties should be looked on as a possible component of the suite of physiological adaptations accompanying the domestication and subsequent evolution of cultivated barley.

## Introduction

The transition from hunting-gathering to agriculture is arguably the most fundamental change in human history [1–6] and the factors responsible for and influencing the domestication of crop plants remain a subject of intense debate. Agriculture began independently in several different parts of the world, one of these locations being the Fertile Crescent of southwest Asia, where the earliest evidence for the appearance of domesticated grain crops–einkorn wheat (*Triticum monococcum* L.), emmer wheat (*T*. *dicoccum* (Schrank) Schübl.) and barley (*Hordeum vulgare* L.)–occurs during the 8^th^ millennium bc [7]. The domesticated version of a plant is distinguished from its wild ancestor by a set of phenotypic features collectively referred to as the domestication syndrome [8,9]. A comparison of cultivated species has shown that a similar set of morphological and physiological traits has been selected during domestication of different crops, these including, for cereals, loss of the natural seed dispersal mechanisms and aids, insensitivity to environmental cues that inhibit germination, and an increase in seed size [9,10]. Most domestication traits are looked on as having zero or low adaptive advantage in the wild but high adaptive advantage in the crop [4], and so are assumed to have been selected as a result of cultivation practices.

Although not conventionally looked on as a domestication trait, it is possible that cultivation also resulted in changes to the water utilization properties of crop plants. Water availability is often the main factor limiting the yield of wild and cultivated grain plants such as wheat and barley in the arid to sub-arid environments of southwest Asia [11,12]. Archaeologically recognisable irrigation systems do not appear until the Early Bronze Age, some 4000–5000 years after the beginning of agriculture, and the extent to which the earliest cultivators compensated for aridity through artificial water management is unknown [13]. If practised, such early watering is likely to have been variable and of low intensity, perhaps involving small-scale earthen channels or watering by hand [14]. Because of their transient nature, direct evidence regarding these early water management practices is difficult to obtain, but water availability during plant growth can be inferred from the stable carbon isotope ratios within tissues [15], including archaeologically charred grain [12, 14, 16–20], and by examination of the weed seeds accompanying crops in archaeobotanical assemblages [21–26]. There is, for example, isotopic evidence suggesting water management or low intensity watering of cereal crops at some Neolithic sites in Western Asia prior to the emergence of fully developed irrigation, though any artificial watering used by these early cultivators may have only partially alleviated the effects of the arid and sub-arid environments within which crops were grown [12,14].

The physiological and genetic response of plants to drought conditions is complex [27–29] and is controlled by a variety of phytohormones including abscisic acid (ABA), cytokinins and ethylene [30]. The initial response to drought stress appears to be mediated by ABA, which is synthesized in roots and transported to other parts of the plant [31,32], resulting in changes in gene expression that adapt the plant to the stress conditions [33]. One way in which phytohormones influence gene expression is via small RNAs including microRNAs (miRNAs), which suppress gene activity by increasing transcript degradation and inhibiting translation [34], and heterochromatic small RNAs (hc-siRNAs), which remodel DNA methylation patterns within the promoters of target genes [35]. Both miRNAs [36] and hc-siRNAs [37] have been implicated in the drought response of cereals. In particular, 24-nucleotide hc-siRNAs have been identified that are present in barley caryopses subject to terminal drought stress but absent in control caryopses grown under normal conditions [37]. The most abundant of these hc-siRNAs was homologous to a region within the promoter of the barley *HvCKX2*.*1* gene, this promoter also containing a binding site for a second, less abundant member of the drought-specific hc-siRNA set. *HvCKX2*.*1* codes for a cytokinin dehydrogenase, a type of enzyme that regulates cytokinin activity by carrying out an oxidoreduction that degrades the target hormone molecules [38]. In seedlings derived from drought stressed barley caryopses, the *HvCKX2*.*1* promoter displays increased methylation, the *HvCKX2*.*1* mRNA content is reduced, and isopentenyladenine and trans-zeatine, which are the target cytokinins for the HvCKX2.1 protein, accumulate [37].

In this paper, we report the diversity of *HvCKX2*.*1* in an extensive range of georeferenced wild barley accessions and cultivated barley landraces and, from the data, suggest that water utilization properties should be looked on as an possible component of the suite of physiological adaptations accompanying the domestication and subsequent evolution of cultivated barley.

## Materials and methods

### Barley accessions

Seeds of 228 barley landraces and 216 wild barley accessions (S1 Table, S2 Table) were obtained from the United States Department of Agriculture–Agricultural Research Service (USDA-ARS) Small Grains Collection (NSGC). Seeds were germinated and seedlings grown in Petri dishes in hydroponic conditions at room temperature (c.22°C). Once the coleoptiles emerged, the seeds were placed on moist filter paper. Fresh leaf material was collected when the seedlings were 21 days old and DNA extracted using the ISOLATE II Plant DNA kit (Bioline).

### DNA sequencing

A 1321 bp segment of the *HvCKX2*.*1* gene, beginning upstream of the start codon and spanning the first and second exons and the intron between these exons, was amplified as two overlapping fragments (amp1 primers: forward 5´–TACCTATACACAAGGTGCCC–3´, reverse 5´–CCCGAGCCCTACATATCAG–3´, 877 bp product, annealing temperature 65°C; amp2 primers: forward 5´–TGGACATGATGTCGCTCGGG–3´, reverse 5´–GATCGACGTCAGACTCACCG–3´, 791 bp, 73°C) and as a single intact product (amp1+2 primers: forward 5´–GAGGGAGTACAGTGTATGCGTATT–3´, reverse 5´–TGATCGACGTCAGACTCACC–3´, 1321 bp, 65°C). PCRs were carried out in a LightCycler480 (Roche) in 20 μl reaction volumes comprising 100 ng DNA extract, 1× SensiFAST SYBR No-ROX PCR master mix (Bioline), 100 nM forward primer, 100 nM reverse primer and PCR grade water. Cycling parameters were: 95°C for 5 min; followed by 35 cycles of 30 s at 95°C, 30 s at the annealing temperature, 60 s at 72°C. Product formation was assayed using the SYBR Green I/HRM Dye detection format (465 nm excitation, 510 nm emission), and melting data were obtained by first cooling the product to 55°C for 30 s and then heating to 99°C with five data acquisitions/°C. PCR products were purified with the High Pure PCR Product Purification Kit (Roche) and sequenced using the BigDye Terminator v3.1 kit chemistry (Applied Biosystems). Standard sequencing reactions comprised 40 ng PCR product, 1× BigDye sequencing buffer, 0.125× BigDye reaction mix, 4 pmoles primer and UltraPure DNase/RNase-free distilled water to give a final volume of 20 μl. Modified reactions comprised 40 ng PCR product, 1× BigDye sequencing buffer, 0.125× BigDye v3.1 reaction mix, 0.0625× dGTP BigDye v3.0 reaction mix, 4 pmoles primer, 0.95 M betaine (Sigma), 5% (v/v) dimethyl sulfoxide (Sigma) and UltraPure DNase/RNase-free distilled water to give a final volume of 20.05 μl. The modified reactions were carried out to avoid early signal loss when sequencing difficult regions such as those with high GC/GT/G content and/or containing small hairpins or other secondary structures. Cycling parameters were: 2 min at 96°C; 35 cycles of 40 s at 96°C, 15 s at 50°C, 4 min at 60°C; with products held at 4°C before purification (Agencourt CleanSEQ; Beckman Coulter) and reading of paired-end sequences by capillary electrophoresis in a 3730 DNA Analyser (Applied Biosystems).

### Data analysis

*HvCKX2*.*1* sequences for individual barley accessions were assembled using Geneious version R10 (https://www.geneious.com, [39]) and multiple alignments of assembled sequences from different accessions were generated by the ClustalW, Muscle and Mafft programs. The consensus sequence of the multiple alignment was identical to the corresponding part of Genbank entry JF495488.1 (*Hordeum vulg*are subsp. *vulgare* cultivar Morex cytokinin oxidase/dehydrogenase [CKX2.1] gene, complete cds). Single nucleotide polymorphisms were identified using the prediction software in Geneious at various settings for maximum variant *p*-value and minimum sequence coverage. Median joining haplotype networks were generated using Network 4 [40] and PopART [41]. Multiple alignment of cytokinin dehydrogenase DNA and protein sequences from different species was carried out online with Clustal Omega [42] at EMBL-EBI. Protein secondary structures were predicted using the garnier tool of EMBOSS [43], operated as a Geneious plug-in. Geographical distribution maps were plotted using ArcMap 10.2.1 of ArcGIS (ESRI. ArcGIS Desktop: Release 10. Redlands, CA: Environmental Systems Research Institute 2011) and correlations between haplotype distribution and modern precipitation data (WorldClim version 2, [44]) were assessed by principal components analysis (PCA) performed with PAST 3.19 [45], and by t-distributed stochastic neighbour embedding (tSNE) and uniform manifold approximation and projection (UMAP) using the Rtsne and umap packages, respectively, of R [46]. A χ^2^ test was performed using GraphPad Prism 8.

## Results

### Diversity of the barley *HvCKX2*.*1* gene and predicted translation product

**Fig 1:**
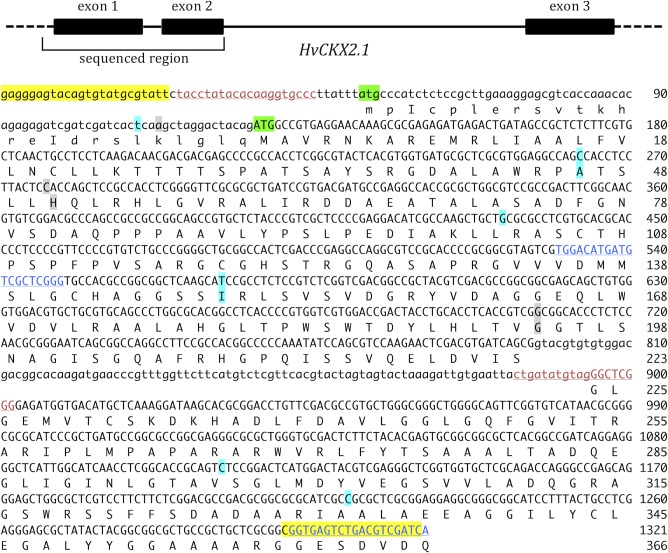
The barley cytokinin dehydrogenase gene *HvCKX2*.*1*. The top panel shows the structure of the gene and the location of the sequenced region. The lower panel shows the sequence of the gene with lower case letters used for the leader sequence and intron, and upper case letters used for the coding region. Primer annealing positions are indicated: amp1 primers, red lettering; amp2 primers, blue lettering; amp1+2 primers, yellow highlight. The possible ATG initiation codons are shown with green highlight. High confidence SNPs and their amino acid substitutions are highlighted in turquoise and medium confidence SNPs and substitutions in grey.

We sequenced *HvCKX2*.*1* in 228 barley landraces and 216 wild barley accessions (S1 Table, S2 Table). Alignment of the sequences revealed multiple variable positions, of which six were identified as high confidence single nucleotide polymorphisms (SNPs) at a maximum variant *p*-value of 10^−9^ and minimum coverage of 393, and a further three were identified as medium confidence SNPs at lower stringency settings (Fig 1, S3 Table). Three of the SNPs, at positions 432, 1112 and 1220 of the amplified region, lie at the third positions within their codons, and do not affect the sequence of the translation product (Table 1). Four other SNPs, at positions 263, 277, 572 and 707, affect the first or second nucleotide of a codon, resulting in the following substitutions: alanine/valine at position 46 of the predicted translation product, histidine/aspartic acid at position 51, isoleucine/threonine at position 149, and glycine/alanine at position 194. The two remaining SNPs, at positions 110 and 113, lie upstream of the initiation codon as listed in the Genbank entry for *HvCKX2*.*1* (accession number JF495488.1), but lie within the coding region of the entry for *HvCKX2*.*1* given in the morexGenes database (sequence ID MLOC_53923.1). The discrepancy is because the morexGenes entry uses an upstream ATG as the initiation codon, increasing the *N*-terminal region of the predicted translation product by 25 amino acids. According to this translation, SNPs 110 and 113 both affect the second position of a codon resulting in leucine/proline and lysine/arginine substitutions, respectively. However, this upstream ATG is not present in the cytokinin dehydrogenase 2 gene of the related grass *Brachypodium distachyon* (S1 Fig), which suggests that for the barley gene the initiation codon used in the Genbank entry is the correct one and that SNPs 110 and 113 do not result in amino acid substitutions.

**Table 1:** SNPs identified at the *HvCKX2*.*1* locus.

Position	Variants	Location in gene	Position in codon	Codon variants	Amino acid variants	Amino acid position in protein
**110**	T, C	upstream	–	–	–	–
**113**	A, G	upstream	–	–	–	–
**263**	C, T	exon 1	second	GCC, GTC	ala, val	46
**277**	C, G	exon 1	first	CAC, GAC	his, asp	51
**432**	G, A	exon 1	third	CTG, CTA	none	102
**572**	T, C	exon 1	second	ATC, ACC	ile, thr	149
**707**	G, C	exon 1	second	GGC, GCC	gly, ala	194
**1112**	C, G	exon 2	third	GTC, GTG	none	296
**1220**	C, T	exon 2	third	GCC, GCT	none	332

Complete data for each of the nine SNP positions were available for 372 accessions. These accessions fall into 14 haplotypes, five of which can be looked on as major haplotypes, comprising 232, 54, 51, 11 and 10 accessions, with the remaining nine haplotypes having four or fewer members each (Table 2, S4 Table). Each of the five major haplotypes is present in wild accessions, and haplotypes 1, 2, 4 and 5 are also represented in the landrace collection. In contrast haplotype 3 is absent in the landraces that we studied. Two other minor haplotypes have multiple members: haplotype 6 with four members, each of these landraces, and haplotype 7 comprising two landraces and one wild accession. The other seven haplotypes have one member each, wild accessions for haplotypes 8 and 11–14, and landraces for haplotypes 9 and 10.

**Table 2:** *HvCKX2*.*1* haplotypes.

Haplotype	SNP positions									Number of accessions		
	110	113	263	277	432	572	707	1112	1220	Wild	Landraces	Total
**1**	T	A	C	C	G	T	G	C	C	67	165	232
**2**	T	A	C	C	G	T	G	G	T	43	11	54
**3**	C	A	T	C	A	C	G	G	T	51	0	51
**4**	T	G	C	C	G	T	G	C	C	2	9	11
**5**	T	A	C	G	G	T	C	C	C	3	7	10
**6**	T	A	C	C	G	T	G	C	T	0	4	4
**7**	T	A	T	C	G	T	G	C	C	1	2	3
**8**	T	A	C	C	G	T	G	G	C	1	0	1
**9**	T	G	C	G	G	T	C	C	C	0	1	1
**10**	T	G	C	C	G	T	G	G	T	0	1	1
**11**	C	A	C	C	G	T	G	G	C	1	0	1
**12**	C	A	C	C	G	T	G	G	T	1	0	1
**13**	C	A	T	C	G	C	G	G	C	1	0	1
**14**	C	A	T	C	G	T	G	G	T	1	0	1

Those landrace haplotypes with more than two members each include accessions with different growth habits, ear row number and caryopsis structure, and the wild haplotypes are similarly variable for growth habit (S5 Table). Comparing the growth habit phenotype of haplotype 3 to that of all other haplotypes by a χ^2^ test yielded a *p* value of 0.4186, demonstrating that haplotype 3 did not significantly associate with a particular growth habit in the wild population. For 72 accessions, missing data prevented identification of the complete haplotype (S4 Table). For 69 of these accessions, replacement of the unidentified nucleotides could give one of the identified haplotypes, with 17 of these, all wild accessions, being possible additional members of haplotype 3. The remaining three of the 72 accessions have partial haplotypes that cannot be extended into either of the 14 identified haplotypes and which therefore represent additional diversity within the *HvCKX2*.*1* gene.

When the amino acid substitutions at positions 46, 51, 149 and 194 of the predicted HvCKX2.1 translation product are considered, there are four protein variants (Table 3). All four variants are present in the wild population but variant B is absent from landraces. Variant B is the only type with a threonine at amino acid position 149, which means that all 200 landraces with complete SNP haplotypes have an isoleucine at this position, whereas this position is threonine for 52 of the 172 wild accessions.

**Table 3:** HvCKX2.1 protein variants.

Variant	Amino acid sequence^a^	DNA haplotype(s)	Number of accessions		
			Wild	Landraces	Total
**A**	ala-his-ile-gly	1, 2, 4, 6, 8, 10, 11, 12	115	190	305
**B**	val-his-thr-gly	3, 13	52	0	52
**C**	ala-asp-ile-ala	5, 9	3	8	11
**D**	val-his-ile-gly	7, 14	2	2	4

^a^The amino acids at positions 46, 51, 149 and 194 are given in order.

**Fig 2:**
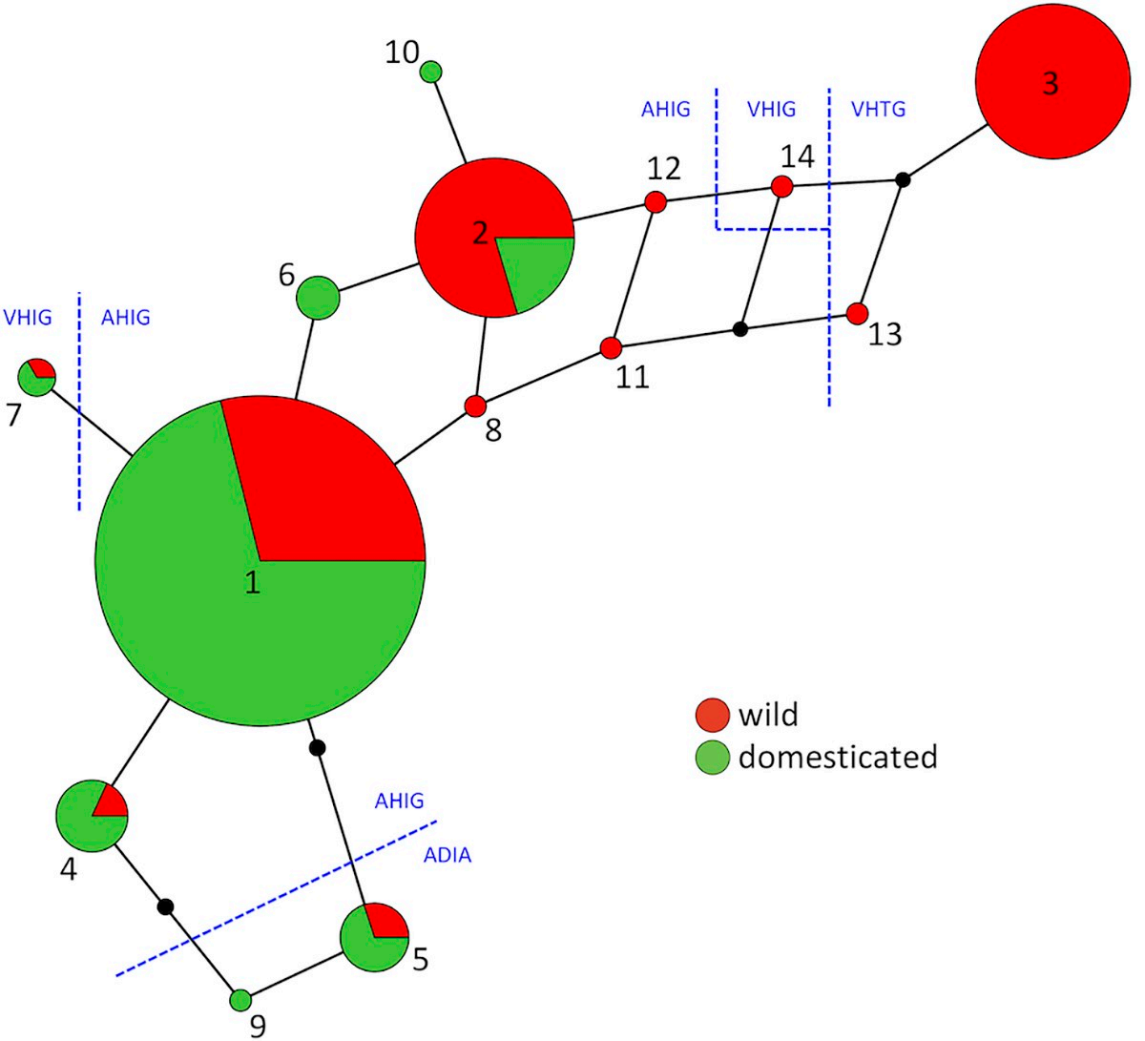
Network displaying the relationships between the fourteen DNA haplotypes of *HvCKX2*.*1*. Node sizes are proportional to numbers of accessions and empty nodes are shown as black dots. The proportion of wild and domesticated accessions for each haplotype are shown in red and green, respectively. The locations within the network of the four protein variants are shown with the amino acid sequences given in the IUPAC single-letter code.

Network analysis (Fig 2) placed major haplotype 1 at a principal position, connected by a maximum of three SNP differences to each of the other haplotypes (haplotypes 2, 4, 6, 8, 10–12) specifying protein variant A (ala-his-ile-gly). Haplotypes 5 and 9, giving protein variant C (ala-asp-ile-ala), form a pair of linked nodes attached to haplotype 1. Protein variant D (val-his-ile-gly) is specified by haplotypes 7 and 14, which occupy different parts of the network, reflecting their dissimilarity at the DNA level (three out of nine SNP differences). Protein variant B (val-his-thr-gly) is coded by the exclusively wild haplotypes 3 and 13, which occupy a distal part of the network.

### Potential effect of *HvCKX2*.*1* gene diversity on the structure of the HvCKX2.1 protein

**Fig 3:**
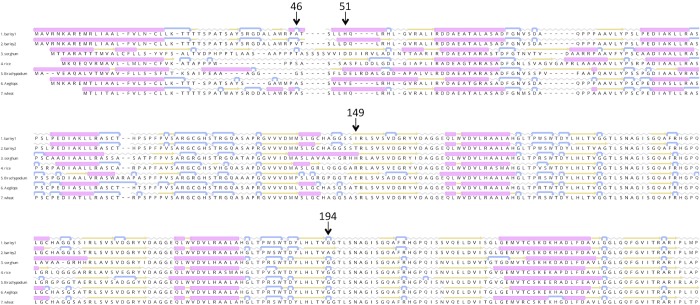
Secondary structure predictions for the barley HvCKX2.1 protein and various other grass cytokinin dehydrogenases. The parts of the protein alignments containing the four substitutions (positions 46, 51, 149 and 194) in HvCKX2.1 are shown. Structural codes: pink barrel, α-helix; yellow arrow, β-strand; blue hooked arrow, turn; grey wavy line, coil. The barley 1 and barley 2 sequences are HvCKX2.1 incorporating the alternative versions of each of the four substitutions. The other sequences are taken from Genbank: sorghum, *Sorghum bicolor* cytokinin dehydrogenase 2, XP_002455003.1; rice, *Oryza sativa japonica* group cytokinin dehydrogenase 2, XP_015629416; Brachypodium, *Brachypodium distachyon* cytokinin dehydrogenase 2-like protein, XP_003564990.3; Aegilops, Aegilops *tauschii* subsp. *tauschii* cytokinin dehydrogenase 2-like protein, XP_020183514.1; wheat, *Triticum aestivum* cytokinin oxidase 2, ADG57787.1.

The potential impact of the four amino acid substitutions on the structure of the barley HvCKX2.1 translation product was assessed by aligning the barley sequence, with and without the substitutions, with the sequences of cytokinin dehydrogenase proteins from related grasses, and then comparing the predicted secondary structures for each of these proteins (Fig 3). The alanine/valine and histidine/aspartic acid substitutions at positions 46 and 51 of the barley protein, respectively, lie within a relatively non-conserved part of the amino acid sequence alignment, although the two positions are alanine and histidine in the most similar wheat protein, and position 46 is alanine in a cytokinin dehydrogenase 2 protein of *Aegilops tauschii*. The two substitutions are predicted to have minor impact on the secondary structure of the barley protein. The alanine/valine substitution affects the length of a short turn in the predicted barley protein, but this turn is not predicted at the equivalent positions of the rice, *B*. *distachyon* and *Ae*. *tauschii* sequences. The histidine/aspartic acid substitution at position 51 affects the length of helical region, which is absent in sorghum and rice and of variable lengths in the other grass proteins. In contrast, the substitutions at positions 149 and 194 of the barley protein lie in regions that display both primary and secondary structural conservation in the grass proteins as a whole. Position 149 is not itself conserved but lies at the *N*-terminus of a predicted β-strand whose length and position is very similar in each sequence. Presence of a threonine at position 149 (the variant absent in landraces) is predicted to stabilise this strand by removing a short turn that is located in the middle of the strand when the isoleucine is present. Position 194 is glycine in each of the other grass proteins, and is located within a 30-amino-acid region that is identical in each of these sequences. The alanine substitution is predicted to move the conserved *C*-terminal position of a β-strand and result in loss of a short conserved turn structure.

**Fig 4:**
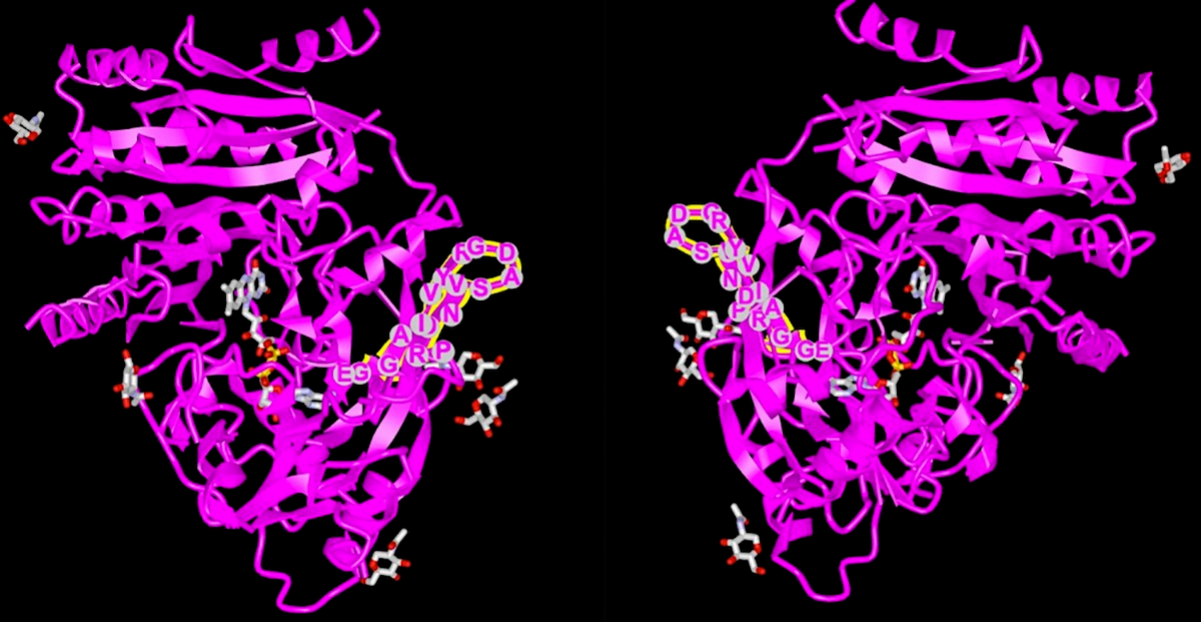
Two views of the X-ray crystallographic structure of a maize cytokinin dehydrogenase protein. The β-strand–turn–β-strand motif that forms a finger on the surface of the protein is highlighted. The structure is PDB ID IW10 and is viewed using the iCnS3D web-base structure viewer at https://www.ncbi.nlm.nih.gov/Structure/icn3d/full.html?complexity=3&buidx=1&showseq=1&mmdbid=29226.

**Fig 5:**
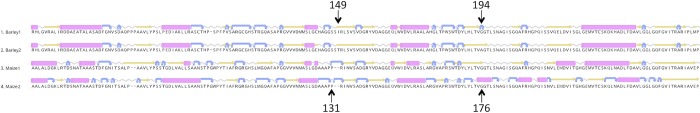
Secondary structure of the FAD binding domains of the barley HvCKX2.1 protein and a maize cytokinin dehydrogenase protein. The Barley 1 and Barley 2 sequences are HvCKX2.1 incorporating the alternative versions of the substitutions at positions 149 and 194. Maize 1 is the secondary structure of a maize cytokinin oxidase/dehydrogenase as predicted from its amino acid sequence, and Maize 2 is the actual secondary structure of this maize cytokinin oxidase/dehydrogenase according to the DSSP analysis of the X-ray crystallographic data of the protein complexed with N6-(3-methoxy-phenyl)adenine (PDB 3DQ0 available at https://www.rcsb.org/structure/3DQ0). Structural codes: pink barrel, α-helix; yellow arrow, β-strand; blue hooked arrow, turn; grey wavy line, coil. Note that the alignment between and the barley and maize amino acid sequences around barley position 149 is slightly different to that shown in S2 Fig.

To obtain additional insights into the potential impact of the amino acid substitutions on the barley protein, the alignment was extended to include a maize cytokinin dehydrogenase whose X-ray crystallographic structure is known [46]. The maize protein consists of a cytokinin-binding domain and a bipartite binding domain for a flavin adenine dinucleotide (FAD) cofactor. The first part of the FAD binding domain is specified by amino acids 40–244 of the maize protein, which correspond to amino acids 54–262 of the barley version (S2 Fig). The alanine/valine and histidine/aspartic acid substitutions at positions 46 and 51 of the barley protein, in a region that displays poor primary and secondary structure conservation in the grass proteins, are therefore immediately upstream of the FAD binding domain. The isoleucine/threonine and glycine/alanine substitutions at positions 149 and 194 both lie within the FAD binding domain, corresponding to positions 131 and 176 of the maize protein. The first of these positions lies within a part of the maize polypeptide that is located at the protein surface, where a β-strand–turn–β-strand motif forms a finger that protrudes slightly away from the main body of the protein (Fig 4). The β-strand–turn–β-strand structure is predicted for the variant of the barley protein with an isoleucine at position 149, but the turn is not predicted when the isoleucine is replaced by threonine (see Fig 3). To test whether this comparison between the actual structure of the maize protein and the predicted structure of the barley protein is valid, we also predicted the secondary structure of the FAD domain of the maize protein from its amino acid sequence. There was good agreement between the predicted and actual structures of the maize protein in the region surrounding position 131, with the prediction identifying the β-strand–turn–β-strand motif at the correct position with only minor conformational differences compared with the actual motif (Fig 5). The accurate prediction of this motif from the maize amino acid sequence, and the agreement between the maize and barley predictions in this region, suggests that the β-strand–turn–β-strand structure is also likely to be a genuine feature of the barley protein when isoleucine is present at position 149, and that this motif might be disrupted by replacement of the isoleucine by threonine in the *HvCKX2*.*1* haplotype that is absent in landraces. The maize structure also includes an asparagine (position 134 of the maize protein) that is the binding site for an *N*-linked *N*-acetylglucosamine sugar residue [47]. The barley protein does not have a potential *N*-linked binding site in this region, but the threonine substitution at barley position 149 would create a potential *O*-linked site. Finally, position 176 of the maize protein (corresponding to the glycine/alanine variation at position 194 of the barley protein) is a glycine located in the same conserved 30-amino-acid region noted above for the other grass proteins, this conserved region including an aspartic acid (position 169 in the maize protein) which is thought to play a critical role as a hydrogen bond acceptor during cytokinin binding [47,48]. In this region, there is poor agreement between the predicted and actual secondary structures of the maize protein, invalidating any further comparisons with the predicted secondary structure of the barley protein.

### Geographical distributions of the *HvCKX2*.*1* haplotypes

**Fig 6:**
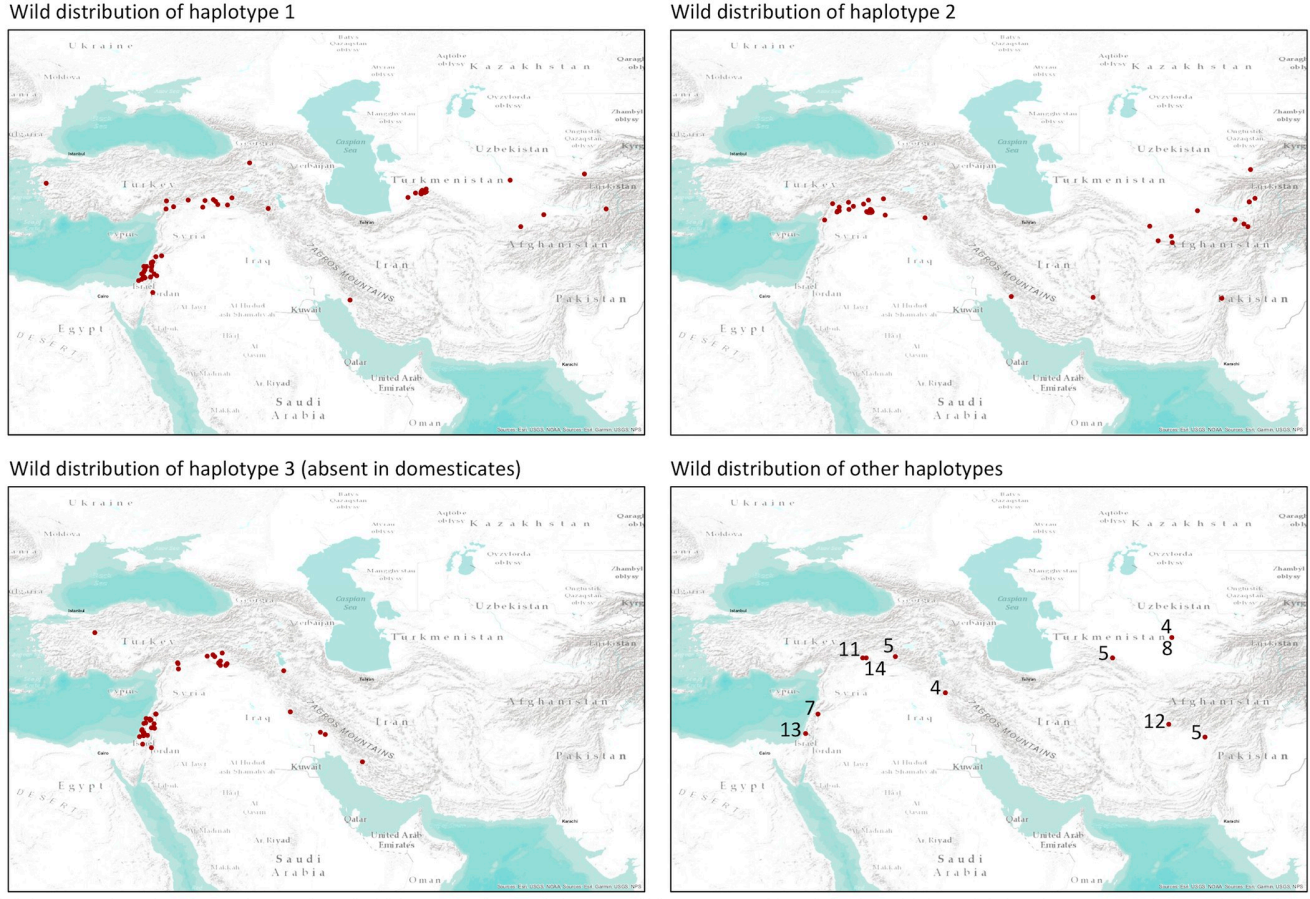
Distributions of wild accessions belonging to different haplotypes. Maps were plotted using ArcMap 10.2.1 of ArcGIS (ESRI. ArcGIS Desktop: Release 10. Redlands, CA: Environmental Systems Research Institute 2011).

We examined the geographical distributions of wild plants of different haplotypes, to assess if the absence of haplotype 3 in landraces could be due to the geographical location(s) of the earliest farming sites in the Fertile Crescent being such that haplotype 3 was not sampled when wild plants were first taken into cultivation. Haplotypes 1–3, which comprise 67, 43 and 51 wild accessions, respectively, have overlapping geographical distributions in the wild population (Fig 6). Wild accessions with haplotype 1 are distributed throughout the Fertile Crescent and are also present in central Asia, including the Balkan region of western Turkmenistan. Haplotype 2 is present in the northern Fertile Crescent and central Asia, but absent from the wild barley population in the southern Levant. Wild accessions with haplotype 3, the haplotype absent in domesticated barley, are distributed throughout the Fertile Crescent. The current distributions within the Fertile Crescent of haplotypes 1 and 3 are therefore very similar, and in the northern part of the arc both distributions include the full range of haplotype 2.

**Fig 7:**
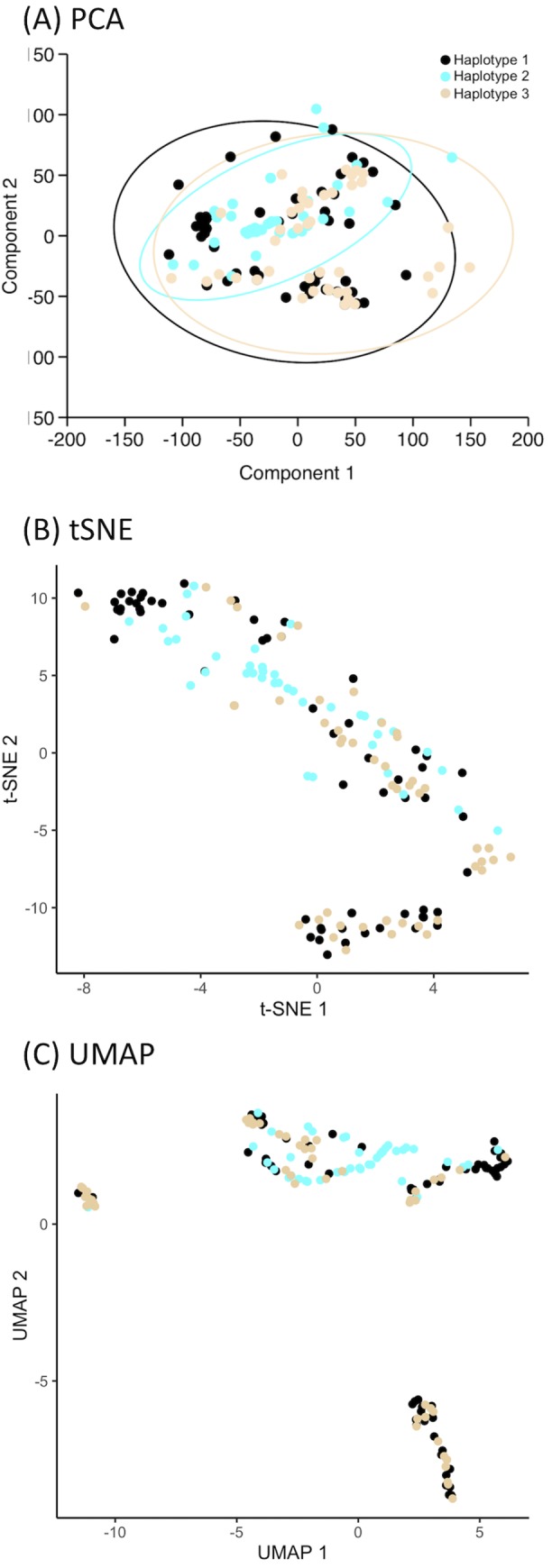
Dimensionality reduction analyses using as input data the combined monthly precipitation amounts (WorldClim version 2) for the collection sites of each wild accession. (A) PCA. Component 1 accounted for 67.8% of the total variability and component 2 accounted for 25.3%. The ellipses indicate the regions within which 95% of the data points for each haplotype are expected to fall. (B) tSNE, run with perplexity = 30, iterations = 2000 and theta = 0.5. (C) UMAP. Black dots and ellipses, haplotype 1; cyan, haplotype 2; orange, haplotype 3.

To explore whether wild accessions belonging to haplotype 3 might respond differently to precipitation, we carried out a PCA using as input data the combined monthly precipitation amounts for the collection sites of each wild accession. When the rainfall data for all months are combined, the resulting plot (Fig 7) shows extensive overlap between the precipitation envelopes for each of the three haplotypes, although wild plants belonging to haplotype 2 occupy a smaller precipitation envelope than either haplotypes 1 or 3, consistent with the less broad geographical distribution of haplotype 2. The envelope for haplotype 3 extends slightly outside of the range of the other two haplotypes in PC1, and excludes an area of the plot occupied by outliers of haplotypes 1 and 2; otherwise, the envelope for haplotype 3 shows no significant difference compared to the combined envelopes for haplotypes 1 and 2. The small differences described above were not apparent when the annual rainfall data for the three haplotypes were analysed by tSNE and UMAP (Fig 7B and 7C).

**Fig 8:**
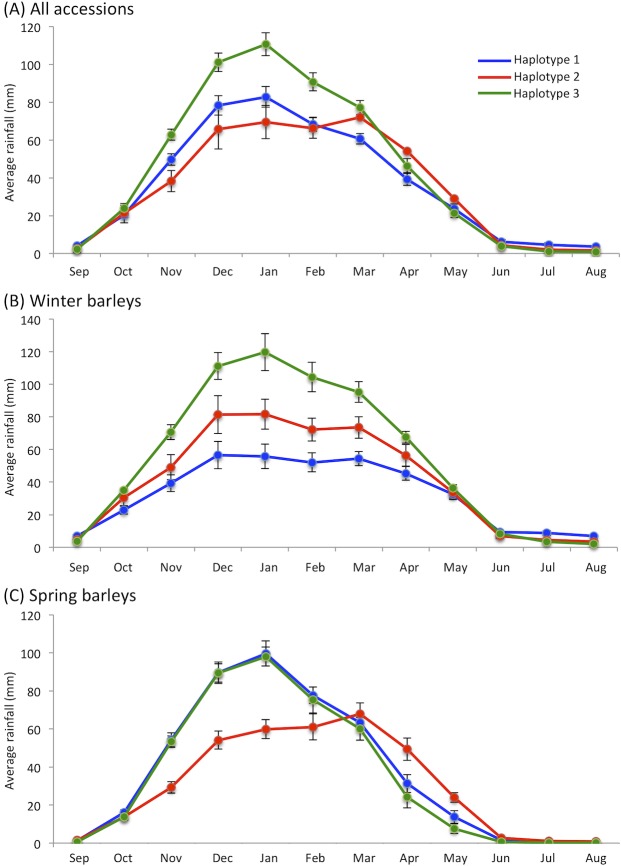
Average monthly precipitation amounts (WorldClim version 2) at the collections sites for wild accessions of haplotypes 1, 2 and 3. (A) All accessions; (B) winter barleys; (C) spring barleys. Blue, haplotype 1; red, haplotype 2; green, haplotype 3. Bars indicate standard error.

To assess if there was any correlation between haplotype and seasonal rainfall patterns, PCA and tSNE were also performed with the rainfall data for different bimonthly periods (S3 Fig, S4 Fig). Again the PCA, but not tSNE, suggested small differences in the rainfall envelope of haplotype 3 compared to the envelopes for haplotypes 1 and 2, at least for the bimonthly periods October/November to March/April. To investigate further, graphs were drawn plotting the average rainfall per month for the collection sites of wild accessions belonging to haplotypes 1, 2 and 3 (Fig 8). The graphs revealed a significant difference in the rainfall data for haplotype 3, these accessions coming from areas with higher rainfall during November to February. This feature was apparent when all the accessions were considered together (Fig 8A) and when the winter barleys were considered on their own (Fig 8B). However, when the springs barleys were examined, there was no significant differences between the plots for haplotypes 1 and 3 (Fig 8C).

## Discussion

We studied the diversity of the barley cytokinin dehydrogenase gene *HvCKX2*.*1* in an extensive range of georeferenced wild barley accessions and cultivated barley landraces. The role of *HvCKX2*.*1* as the target of the most abundant drought-responsive hc-siRNAs in barley caryopses, and the reduced *HvCKX2*.*1* expression that occurs in seedlings derived from drought stressed plants, indicates that this gene contributes to the water utilization properties of barley plants. Our results show that cultivated barley landraces lack one of the five major haplotypes of the *HvCKX2*.*1* gene present in the wild population, resulting in the absence in landraces of a version of the cytokinin dehydrogenase protein with a threonine rather than isoleucine at position 149 of the predicted translation product. This position lies within the FAD binding domain of the protein, comparison with the X-ray structure of a maize cytokinin dehydrogenase suggesting that the isoleucine/threonine substitution affects the conformation of a finger motif that projects from the surface of the protein. According to secondary structure prediction, this finger motif is conserved in the isoleucine version of the barley protein, but is disrupted by the threonine substitution, resulting in loss of the turn linking the two β-strands of the finger. Additionally, the maize motif is linked to an *N*-acetylglucosamine sugar residue which would be absent from the isoleucine version of the barley protein because this sequence lacks a potential glycosylation site, though such a site would be created by the threonine substitution. Although there are no published data reporting a role for the finger motif in the function of the maize protein, the predicted structural changes that we describe make it possible that the presence of threonine rather than isoleucine at position 149 results in a change in the properties of the HvCKX2.1 protein.

There are several possible explanations for the apparent absence of haplotype 3 in landraces. This first is that this is simply due to sampling bias that occurred when we assembled our landrace collection. However, the likelihood of haplotype 3 being excluded by sampling bias is low: if, in reality, haplotype 3 is present in landraces at a frequency of 29.65% (the frequency of this haplotype in the wild accessions) then the probability of haplotype 3 being absent in a random collection of 200 landraces is 2.84×10^−31^. Our landrace collection had a broad geographical distribution (see S1 Table) and hence was unlikely to be so non-random as to bias this probability to the extent that haplotype 3 was missed due to sampling effects.

A second possibility is that the absence of haplotype 3 in landraces is due to sampling bias that occurred when the first wild barley plants were taken into cultivation. If barley was initially domesticated from a wild population that contained relatively few genotypes then it is possible that haplotype 3 was missed purely by chance, and hence never made its way into the crop. In our view, two factors reduce the likelihood of this scenario. First, in the modern wild population, the geographical distributions of haplotypes 1 and 3 are very similar and both encompass the full range of haplotype 2. If the modern phylogeography reflects the haplotype frequency and distribution when barley was first taken into cultivation, then this would appear to mitigate against the possibility that the early farmers, purely by chance, only domesticated wild plants belonging to haplotypes 1 and 2, to which the majority of the landraces belong, when haplotype 3 plants were growing in similar areas. The second argument which in our view makes its unlikely that haplotype 3 was excluded by chance from the crop is the evidence from genome-wide studies that cultivated barley emerged as a genetic mosaic of wild source populations, with the diversity of the crop established in part by hybridization between early cultivated forms and various wild populations [49,50]. Exclusion of haplotype 3 purely by chance therefore requires not only the absence of this haplotype among the initial set(s) of plants taken into cultivation, but also the absence of haplotype 3 in any of the wild populations with which the early crop subsequently hybridized.

From the above considerations it seems plausible that the absence of haplotype 3 in landraces is due to these plants being less suited to the artificial conditions associated with cultivation. Cytokinin dehydrogenases are one of a number of enzyme families that participate in the cytokinin signalling pathway of plants [51], this pathway regulating diverse physiological processes involved in plant growth, development and the response to stresses such as drought and heat. Although *HvCKX2*.*1* has been highlighted as the regulatory target for the most abundant hc-siRNAs in barley caryopses subject to terminal drought stress [37], this does not preclude the possibility that the HvCKX2.1 protein has other, as yet undetected functions in those parts of the cytokinin signalling pathway that operate in developing caryopses and/or in seedlings up to 12–24 hours after imbibition, these being the growth stages when *HvCKX2*.*1* RNA is present in plant tissue [37]. If the protein has other such roles, then any one of these, or a combination, could underlie the absence of haplotype 3 in landraces. However, we believe that it is reasonable based on what is known about the role of *HvCKX2*.*1* in the drought response to propose as a working hypothesis that the particular aspect of cultivation that mitigates against haplotype 3 relates in some way to water utilization. The rainfall analysis would appear to support this hypothesis, by suggesting that there are differences in the preferred precipitation patterns for plants of different haplotypes in the natural environment, manifested most clearly by the significantly higher rainfall at the collection sites of haplotype 3 plants during November to March (Fig 8), which includes the period when grain from plants with a winter growth habit is undergoing germination and early seedling growth.

The hypothesis that haplotype 3 plants are less suited to the artificial hydrological conditions associated with cultivation is prompted by the genetic data that we report in this paper, but comparison between the genetic data and environmental factors can only ever provide indirect support for such a hypothesis. Confirmation of the hypothesis would require detailed functional studies aimed at discerning some difference between the physiological properties of plants belonging to haplotype 3 (or more specifically to plants whose HvCKX2.1 protein contains a threonine rather than isoleucine at position 149) and plants carrying other versions of *HvCKX2*.*1*. Transgenic experiments or gene editing could be used to ensure that the properties of different *HvCKX2*.*1* variants are examined in a uniform genetic background. Such studies would be complex, as the precise nature of any phenotypic change cannot be predicted and could be subtle, but the presence of *HvCKX2*.*1* mRNA in developing caryopses and in seedlings up to 12–24 hours after imbibition [37] suggests that the altered phenotype is likely to be expressed during grain filling and/or germination. Sequencing of *HvCKX2*.*1* transcripts might also be carried out to check if there are any differences in splice site selection and the usage of transcription start sites and polyadenylation sites in wild and domesticated plants of different haplotypes.

## Conclusion

The traditionally recognised traits characterizing the domestication syndrome for grain crops such as barley include loss of the natural seed dispersal mechanisms, increase in seed size, and insensitivity to environmental cues that inhibit germination [8–10]. It has been suggested, however, that domestication of wild grasses was also accompanied by selection for physiological changes driven by early cultivation practices [52,53]. Our results highlight the possibility that one of these practices was water management, and that water utilization properties should be looked on as a possible component of the suite of physiological adaptations accompanying the domestication of barley and, by implication, other grain crops that were domesticated in arid or semi-arid environments. By raising the possibility that genetic adaptation occurred in response to the artificial hydrological conditions associated with cultivation, our results also emphasise the important role that water availability played during the emergence of agriculture in the Fertile Crescent, and indicate that the development of crop husbandry techniques able to mitigate against water stress could have been a major factor in ensuring the sustainability of early cultivation in the region.

## Supporting information

S1 Table Barley landraces used in this study. (XLSX)

S2 Table Wild barley accessions used in this study. (XLSX)

S3 Table Positions of SNPs identified at difference confidence settings. (XLSX)

S4 Table Haplotype identities for the landraces and wild barley accessions. (XLSX)

S5 Table Phenotypes of accessions belonging to different HvCKX2.1 haplotypes. (XLSX)

S1 Fig Alignment between the upstream regions of the *Brachypodium distachyon* cytokinin dehydrogenase 2-like gene and the barley *HvCKX2*.*1* gene. Nucleotides in the upstream regions are in lower case and those in the coding regions in uppercase, with nucleotide identities indicated by asterisks. Potential initiation codons are highlighted in green. The *Brachypodium* gene is Genbank accession number XM_003564942.3 and the barley gene is accession number JF495488.1.
(TIFF)

S2 Fig Alignment between different cytokinin dehydrogenase genes. The location of the FAD binding domain is shown in red, based on the sequence of the maize protein. Amino acids highlighted in green in the barley sequence are those substituted in the different HvCKX2.1 protein variants. Asterisks indicate positions where the amino acid is identical in each sequence; colons indicate positions occupied by amino acids with strongly similar properties (>0.5 in the Gonnet point accepted mutation [PAM] 250 matrix); periods indicate positions occupied by amino acids with weakly similar properties (<0.5 and >0 in the Gonnet PAM 250 matrix). The barley sequence is the HvCKX2.1 consensus sequence as determined in this study (identical with Genbank JF495488.1). The other sequences are taken from Genbank: maize, *Zea mays* cytokinin oxidase 1, accession number ONM29023.1; sorghum, *Sorghum bicolor* cytokinin dehydrogenase 2, XP_002455003.1; rice, *Oryza sativa japonica* group cytokinin dehydrogenase 2, XP_015629416; Brachypodium, *Brachypodium distachyon* cytokinin dehydrogenase 2-like protein, XP_003564990.3; Aegilops, *Aegilops tauschii* subsp. *tauschii* cytokinin dehydrogenase 2-like protein, XP_020183514.1; wheat, *Triticum aestivum* cytokinin oxidase 2, ADG57787.1.
(TIFF)

S3 Fig PCAs using as input data the bimonthly precipitation amounts (WorldClim version 2) for the collection sites of each wild accession. The ellipses indicate the regions within which 95% of the data points for each haplotype are expected to fall. Black dots and ellipses, haplotype 1; cyan, haplotype 2; orange, haplotype 3.
(TIFF)

S4 Fig tSNE using as input data the bimonthly precipitation amounts (WorldClim version 2) for the collection sites of each wild accession. tSNE was run with perplexity = 30, iterations = 2000 and theta = 0.5. Black dots and ellipses, haplotype 1; cyan, haplotype 2; orange, haplotype 3.
(TIFF)
