# Supplementary figures and images for: Diversity of a cytokinin dehydrogenase gene in wild and cultivated barley
Source: PLoS One. 2019 Dec 5;14(12):e0225899. doi: 10.1371/journal.pone.0225899 (PMC6894797; doi:10.1371/journal.pone.0225899)

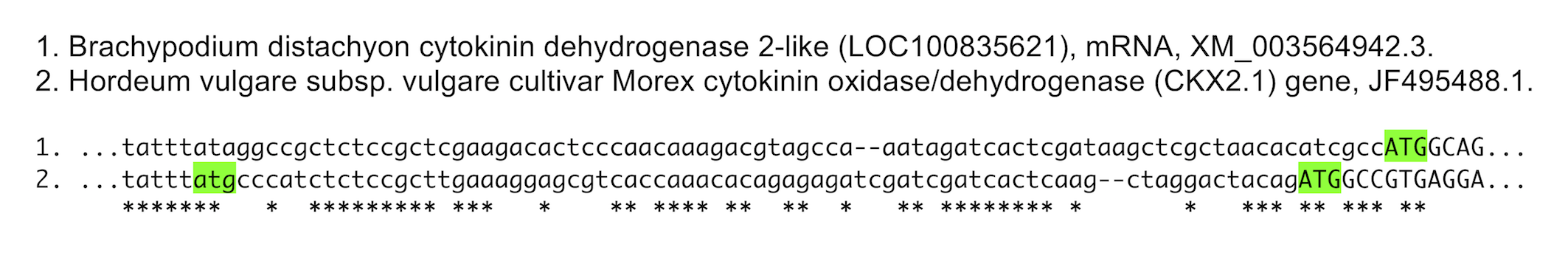

Supplement: S1 Fig — Nucleotides in the upstream regions are in lower case and those in the coding regions in uppercase, with nucleotide identities indicated by asterisks. Potential initiation codons are highlighted in green. The Brachypodium gene is Genbank accession number XM_003564942.3 and the barley gene is accession number JF495488.1. (TIFF) [file pone.0225899.s006.tiff]

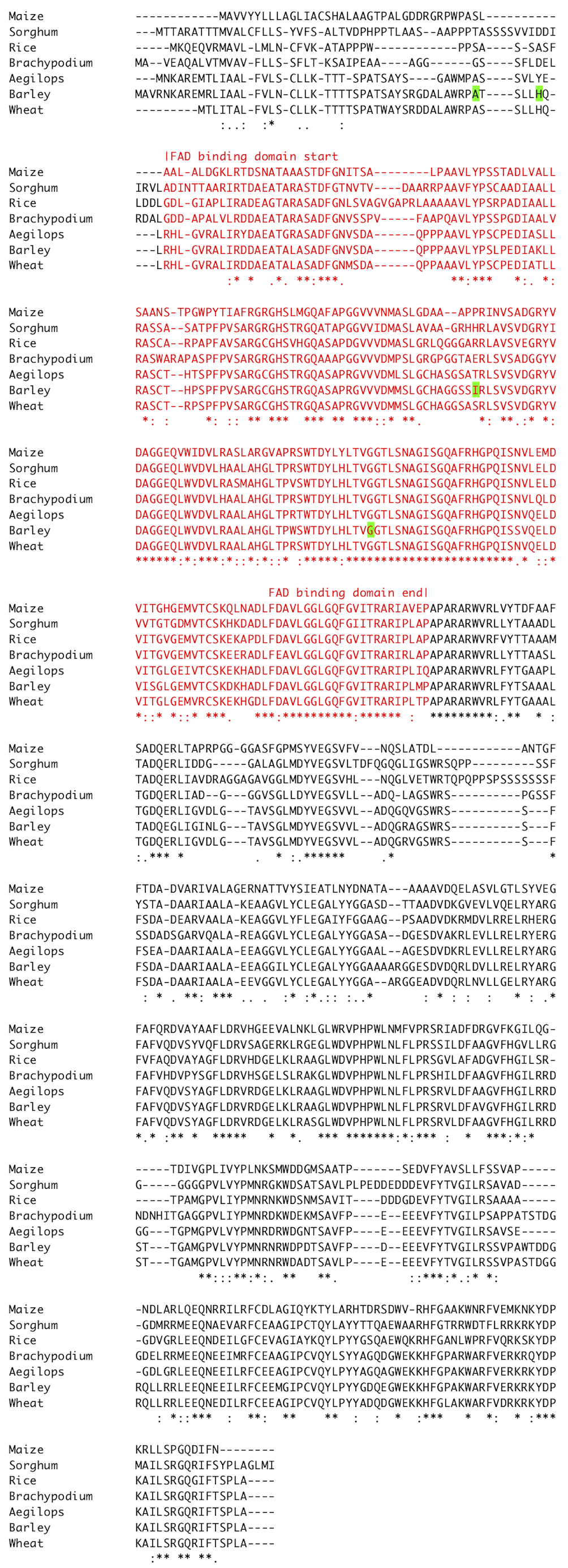

Supplement: S2 Fig — The location of the FAD binding domain is shown in red, based on the sequence of the maize protein. Amino acids highlighted in green in the barley sequence are those substituted in the different HvCKX2.1 protein variants. Asterisks indicate positions where the amino acid is identical in each sequence; colons indicate positions occupied by amino acids with strongly similar properties (>0.5 in the Gonnet point accepted mutation [PAM] 250 matrix); periods indicate positions occupied by amino acids with weakly similar properties (<0.5 and >0 in the Gonnet PAM 250 matrix). The barley sequence is the HvCKX2.1 consensus sequence as determined in this study (identical with Genbank JF495488.1). The other sequences are taken from Genbank: maize, Zea mays cytokinin oxidase 1, accession number ONM29023.1; sorghum, Sorghum bicolor cytokinin dehydrogenase 2, XP_002455003.1; rice, Oryza sativa japonica group cytokinin dehydrogenase 2, XP_015629416; Brachypodium, Brachypodium distachyon cytokinin dehydrogenase 2-like protein, XP_003564990.3; Aegilops, Aegilops tauschii subsp. tauschii cytokinin dehydrogenase 2-like protein, XP_020183514.1; wheat, Triticum aestivum cytokinin oxidase 2, ADG57787.1. (TIFF) [file pone.0225899.s007.tiff]

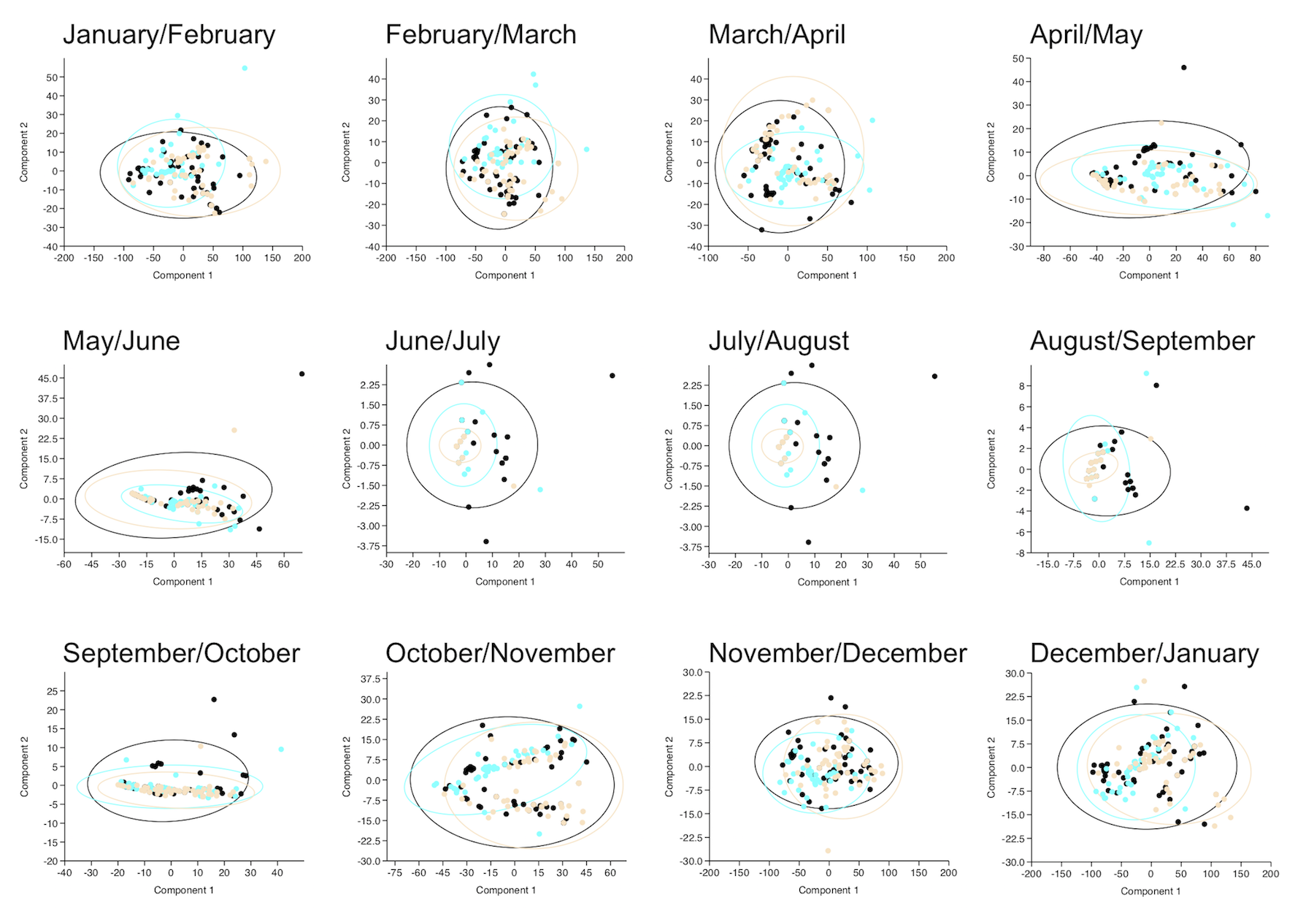

Supplement: S3 Fig — The ellipses indicate the regions within which 95% of the data points for each haplotype are expected to fall. Black dots and ellipses, haplotype 1; cyan, haplotype 2; orange, haplotype 3. (TIFF) [file pone.0225899.s008.tiff]

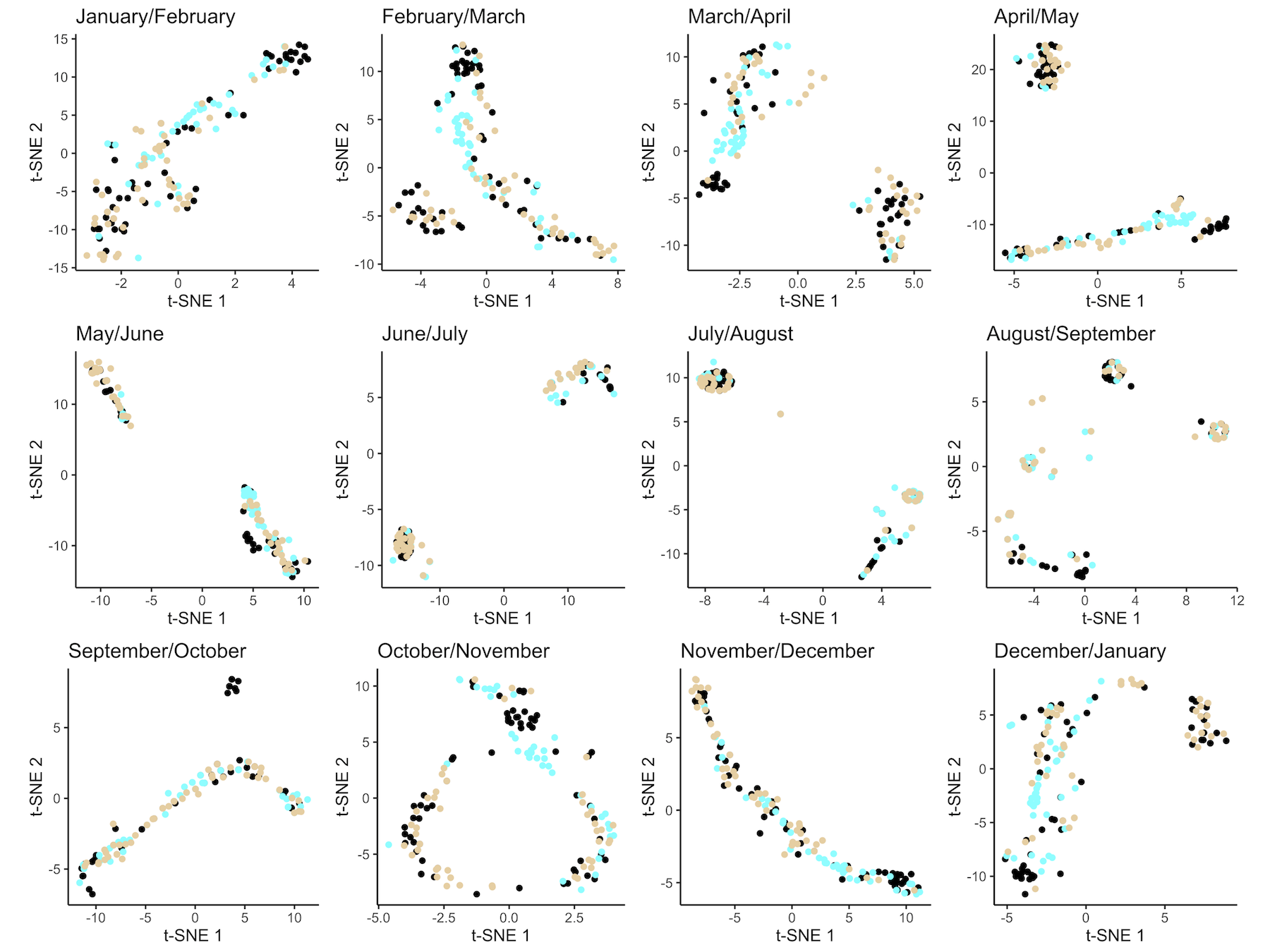

Supplement: S4 Fig — tSNE was run with perplexity = 30, iterations = 2000 and theta = 0.5. Black dots and ellipses, haplotype 1; cyan, haplotype 2; orange, haplotype 3. (TIFF) [file pone.0225899.s009.tiff]
